# Supplementary material for: Integrated Purification and Formulation of an Active Pharmaceutical Ingredient via Agitated Bed Crystallization and Fluidized Bed Processing
Source: Pharmaceutics. 2022 May 14;14(5):1058. doi: 10.3390/pharmaceutics14051058 (PMC9145956; doi:10.3390/pharmaceutics14051058)
Supplement: Supplementary file 1 [file pharmaceutics-14-01058-s001.zip › pharmaceutics-1672329-supplementary.pdf]

Supplementary Materials

Integrated Purification and Formulation of an Active Pharmaceutical Ingredient *via* Agitated Bed Crystallization and Fluidized Bed Processing

Michael W. Stocker[1], Matthew J. Harding[1,2],Valerio Todaro[3], Anne Marie Healy[3], Steven Ferguson [1,2,4,5]*

1. School of Chemical and Bioprocess Engineering, University College Dublin, Dublin 4, Ireland

2. I-form, The SFI Research Centre for Advanced Manufacturing, School of Chemical and Bioprocess Engineering, University College Dublin, Dublin 4, Ireland

3. SSPC, The SFI Research Centre for Pharmaceuticals, School of Pharmacy and Pharmaceutical Sciences, Panoz Institute, Trinity College Dublin, Dublin 2, Ireland

4. SSPC, The SFI Research Centre for Pharmaceuticals, School of Chemical and Bioprocess Engineering, University College Dublin, Dublin 4, Ireland

5. National Institute for Bioprocess Research and Training, 24 Foster's Ave, Belfield, Blackrock, Co. Dublin A94 X099, Ireland

DLE Final Equations for Combinations of Categoric Factors

Binder: PVP

Solvent: Ethanol

$$\begin{aligned}\text{DLE} = 111.52 &- (0.98 \cdot \text{Inlet Temperature}) - (1.61 \cdot \text{Binder Mass}) \\ &- (1.14 \cdot \text{Solvent Volume}) \\ &+ (0.02 \cdot \text{Inlet Temperature} \cdot \text{Solvent Volume}) \\ &+ (0.12 \cdot \text{Binder Mass} \cdot \text{Solvent Volume})\end{aligned}$$

Binder: PVP

Solvent: Methanol

$$\begin{aligned}\text{DLE} = 202.32 &- (2.06 \cdot \text{Inlet Temperature}) - (9.58 \cdot \text{Binder Mass}) \\ &- (1.14 \cdot \text{Solvent Volume}) \\ &+ (0.02 \cdot \text{Inlet Temperature} \cdot \text{Solvent Volume}) \\ &+ (0.12 \cdot \text{Binder Mass} \cdot \text{Solvent Volume})\end{aligned}$$

Binder: HPMC

Solvent: Ethanol

$$\begin{aligned}\text{DLE} = 98.477 &- (0.98 \cdot \text{Inlet Temperature}) - (1.61 \cdot \text{Binder Mass}) \\ &- (0.85 \cdot \text{Solvent Volume}) \\ &+ (0.02 \cdot \text{Inlet Temperature} \cdot \text{Solvent Volume}) \\ &+ (0.12 \cdot \text{Binder Mass} \cdot \text{Solvent Volume})\end{aligned}$$

Binder: HPMC

Solvent: Methanol

$$\begin{aligned}\text{DLE} = 171.43 &- (2.06 \cdot \text{Inlet Temperature}) - (9.58 \cdot \text{Binder Mass}) \\ &- (0.85 \cdot \text{Solvent Volume}) \\ &+ (0.02 \cdot \text{Inlet Temperature} \cdot \text{Solvent Volume}) \\ &+ (0.12 \cdot \text{Binder Mass} \cdot \text{Solvent Volume})\end{aligned}$$

DoC final equations for combinations of categoric factors

Binder: PVP

Solvent: Ethanol

$$\mathrm{DLE} = 298.63 - (2.83 \cdot \mathrm{Inlet\ Temperature}) - (38.72 \cdot \mathrm{Binder\ Mass}) - (0.42 \cdot \mathrm{Solvent\ Volume}) + (0.18 \cdot \mathrm{Binder\ Mass} \cdot \mathrm{Solvent\ Volume})$$

Binder: PVP

Solvent: Methanol

$$\mathrm{DLE} = 35.55 - (1.04 \cdot \mathrm{Inlet\ Temperature}) - (23.05 \cdot \mathrm{Binder\ Mass}) - (0.42 \cdot \mathrm{Solvent\ Volume}) + (0.18 \cdot \mathrm{Binder\ Mass} \cdot \mathrm{Solvent\ Volume})$$

Binder: HPMC

Solvent: Ethanol

$$\mathrm{DLE} = 270.88 - (2.83 \cdot \mathrm{Inlet\ Temperature}) - (22.86 \cdot \mathrm{Binder\ Mass}) - (0.82 \cdot \mathrm{Solvent\ Volume}) + (0.18 \cdot \mathrm{Binder\ Mass} \cdot \mathrm{Solvent\ Volume})$$

Binder: HPMC

Solvent: Methanol

$$\mathrm{DLE} = 27.93 + (1.04 \cdot \mathrm{Inlet\ Temperature}) - (7.19 \cdot \mathrm{Binder\ Mass}) - (0.82 \cdot \mathrm{Solvent\ Volume}) + (0.18 \cdot \mathrm{Binder\ Mass} \cdot \mathrm{Solvent\ Volume})$$
